# Cerebral Metabolite Concentrations Are Associated With Cortical and Subcortical Volumes and Cognition in Older Adults

**DOI:** 10.3389/fnagi.2020.587104

**Published:** 2021-02-03

**Authors:** John B. Williamson, Damon G. Lamb, Eric C. Porges, Sarah Bottari, Adam J. Woods, Somnath Datta, Kailey Langer, Ronald A. Cohen

**Affiliations:** ^1^Center for Cognitive Aging and Memory, Clinical Translational Research Program, College of Medicine, University of Florida, Gainesville, FL, United States; ^2^Center for OCD and Anxiety Related Disorders, Department of Psychiatry, McKnight Brain Institute, College of Medicine, University of Florida, Gainesville, FL, United States; ^3^Department of Clinical and Health Psychology, College of Public Health and Health Professions, University of Florida, Gainesville, FL, United States; ^4^Brain Rehabilitation Research Center, Malcom Randall VA Medical Center, Gainesville, FL, United States; ^5^Department of Biostatistics, College of Public Health and Health Professions, University of Florida, Gainesville, FL, United States

**Keywords:** MRS, MRI, brain volume, N-acetylaspartate, healthy aging, Alzheimer’s disease, cognitive, choline

## Abstract

**Background:**

Cerebral metabolites are associated with different physiological processes in brain aging. Cortical and limbic structures play important roles in cognitive aging; however, the relationship between these structures and age remains unclear with respect to physiological underpinnings. Regional differences in metabolite levels may be related to different structural and cognitive changes in aging.

**Methods:**

Magnetic resonance imaging and spectroscopy were obtained from 117 cognitively healthy older adults. Limbic and other key structural volumes were measured. Concentrations of N-acetylaspartate (NAA) and choline-containing compounds (Cho) were measured in frontal and parietal regions. Neuropsychological testing was performed including measures of crystallized and fluid intelligence and memory.

**Results:**

NAA in the frontal voxel was associated with limbic and cortical volumes, whereas Cho in parietal cortex was negatively associated with hippocampal and other regional volumes. Hippocampal volume was associated with forgetting, independent of age. Further, parietal Cho and hippocampal volume contributed independent variance to age corrected discrepancy between fluid and crystallized abilities.

**Conclusion:**

These findings suggest that physiological changes with age in the frontal and parietal cortices may be linked to structural changes in other connected brain regions. These changes are differentially associated with cognitive performance, suggesting potentially divergent mechanisms.

## Introduction

Brain atrophy is well established in the context of normal aging. Quantitative longitudinal analysis of brain volumes provides compelling evidence that regionally specific volume loss occurs with advancing age in most people (e.g., limbic and hippocampal regions; Lim et al., 1990). In a longitudinal assessment of 1,172 older healthy adults, after 65 years old, the highest rates of regional gray matter loss were found in frontal and parietal regions, temporal cortex and hippocampus (Crivello et al., 2014).

The functional significance of these subtle brain volume changes over short time frames may be minimal. Yet, cognitive performance tends to correspond with overall cerebral volume, as well as specific cortical and subcortical volume depending on function (Fletcher et al., 2018). Greater than normal brain atrophy is often associated with comparative reductions in cognitive functions. In addition, among people who develop cognitive impairment, atrophy in the temporal lobes is detectable before manifestation of cognitive decline (Pacheco et al., 2015). Functional imaging and cognitive testing corroborate these findings, showing changes in aging, particularly in prefrontal and limbic brain regions (Grady et al., 2003; Rodriguez-Aranda and Sundet, 2006; Yin et al., 2016). Physiological relationships to these regional changes in structure are unclear. Understanding physiological relationships between these hierarchically linked structures may provide insight into the process of cognitive and brain aging.

Alterations in cerebral metabolites occur in neurodegenerative disorders as well as in the context of normal aging (Kakimoto et al., 2016). Cerebral metabolite concentrations may be relevant as a marker of brain health and the presence of pathophysiological process. Several of these metabolites can be reliably quantified by magnetic resonance spectroscopy (MRS) and may be relevant to brain aging including N-acetylaspartate (NAA), myo-inositol (MI), choline-containing compounds (Cho), glutamate and glutamine (Glx), and creatine and phosphocreatine (Cr). Each of these cerebral metabolites plays a role in brain physiology. NAA is a precursor for certain neuronal peptides and a source for acetate for lipid and myelin synthesis in glial cells. It contributes to energy production from glutamate in neuronal mitochondria and helps to regulate fluid balance through neuronal osmosis. Its potential clinical significance arises from the fact that NAA has been shown to be important in neuronal function, Singhal et al. (2017) such that decreased NAA has been shown to be related to neuronal damage (Wagner et al., 2013) and reduced structural brain volume in neurological disease (Cohen et al., 2010). Further, decreased levels of NAA are associated with impaired neuronal function, cognition (Paul et al., 2016) and a variety of brain disorders that affect cognition (Penner et al., 2015; Cysique et al., 2018). MI plays an important role in insulin signaling (Jones and Varela-Nieto, 1999), intracellular calcium regulation (Berridge and Irvine, 1989), the cytoskeleton assembly (Janmey et al., 1999), fat breakdown (Plows et al., 2017), and cell membrane maintenance (Shewan et al., 2011). MI has been shown to be sensitive to glial function and osmosis, and therefore its concentrations in the brain are often elevated in the context of neuroinflammation (Burger et al., 2018). Finally, Cho concentrations also have been reported to be elevated in clinical populations (Cohen et al., 2010) and have been linked to inflammation (Kocevar et al., 2017). The components of Glx include glutamine and glutamate. Glutamine and glutamate are found in glia and neurons and are important in normal function of neurons. Glutamate is the most abundant excitatory neurotransmitter in the brain and is important in a variety of neuronal functions (Ramadan et al., 2013). The components of Cr in the MRS signal include creatine, a nitrogen-based organic acid that provides energy, and phosphocreatine, an energy reserve in the brain (Valenzuela et al., 2003).

Each of these cerebral metabolites has been implicated in diseases that affect the brain (Foong et al., 1999; Kizu et al., 2004; Zhang et al., 2009; Ahluwalia et al., 2013). With advancing age, there are changes in cerebral metabolite levels. NAA has been reported to decrease with age; however, this may be regionally specific. In a recent study measuring NAA in posterior cingulate cortex, it was reported that NAA concentrations did not decrease with age, but rather the ratio of NAA/Cr (combined creatine and phosphocreatine) decreased (Suri et al., 2017). Historically, many metabolites were considered in ratio to Cr; however, interpretation of this approach in the context of aging research may be challenging as reports exist demonstrating Cr concentrations decrease in older populations (Suri et al., 2017). Futher complicating the interpretation, in the hippocampus, bilateral reductions in NAA and in NAA/Cr have been demonstrated.

This study was designed to examine relationships between cerebral metabolites, brain volumes and cognitive performance. NAA, MI, Cho, Glx, and Cr play important roles in maintaining healthy brain function. There is heterogeneity of distribution of change in these metabolites with age depending on region of assessment (Schmitz et al., 2018). Further, there is heterogeneous regional volumetric changes in brain morphology with age, along with alterations that occur in cognitive abilities with age (Driscoll et al., 2009). These differences may reflect different physiological processes associated with brain aging. Understanding how regional differences in metabolic features are related to regional changes in key structural volumes important in cognitive performance may suggest possible pathophysiological processes associated with aging that predict vulnerability to cognitive decline. There is a paucity of this sort of information in the literature. To date, there are no investigations that have examined the relationship between cerebral metabolite concentrations and age-associated brain volumetric indices in the context of normal brain and cognitive aging in specific regions.

Normal aging is a heterogenous group. In a cross-sectional sample of cognitively normal older individuals, in addition to the effects of normal aging, there are likely to be individuals who later go on to develop mild cognitive impairment or Alzheimer’s disease. There may be increased sensitivity in predicting who may develop MCI and AD through the use of analyses of regional metabolites, atrophy and cognitive performance.

A regional analysis may be particularly valuable due to the interconnected organization of brain regions that decline in aging. We hypothesized that cerebral metabolite concentrations in the prefrontal region would be associated with interconnected subcortical brain structures (e.g., hippocampus and amygdala). Understanding that Alzheimer’s changes tend to present in parietal cortex and hippocampus and may present subclinically in older adults with normal cognition, it may be that metabolic changes in parietal lobe are associated with hippocampal changes and cognitive performance. We also examined the relationship of frontal and parietal metabolites with overall cortical and subcortical gray and white matter, though we did not have specific hypotheses with these regions. Abnormalities in interconnected systems may suggest a pathophysiological process underlying regional deterioration and specific patterns of cognitive performance changes seen in brain aging (e.g., decreases in processing speed and memory efficiency). This could be important both in suggesting mechanism and establishing new intervention targets.

## Materials and Methods

### Participants

One hundred seventeenth older adults (69 women) were recruited from the North-Central Florida community. The racial composition of the sample was 93 percent Caucasian, 4 percent African American, 1 percent Hispanic, and 2 percent other. Participants responded to advertisements for a study on healthy aging. Participants had a mean age of 71.3 years (SD = 10.01 years) and ranged in age from 43–89 years. The average education level was 16.12 years (SD = 2.70), and the average Montreal Cognitive Assessment (MoCA) score was 25.63 (SD = 2.62). All participants provided written informed consent prior to enrollment. All study procedures were approved by the University of Florida Institutional Review Board prior to the start of the study. Exclusionary criteria included pre-existing significant neurological (an isolated concussion was not an exclusion, whereas history of epilepsy, multiple sclerosis, moderate or severe TBI, or stroke would be) or psychiatric brain disorders (history of depression was not an exclusion, whereas history of schizophrenia, schizoaffective disorder, obsessive compulsive disorder and similar conditions would be), MRI contraindications, or diagnosis with a neurodegenerative brain disease such as Alzheimer’s disease. See Table 1 for demographic and cardiovascular disease risk history (hypertension, diabetes, hypercholesterolemia, current smoking status, and history of myocardial infarct).

**TABLE 1:** Demographics and health characteristics.

Age (mean ± SD)	Sex	Education (mean ± SD)	Hypertension (frequency)	Diabetes (frequency)	High Cholesterol (frequency)	Current cigarette use (frequency)	History of myocardial infarct (frequency)
71 ± 10	69 women	16 ± 2.7 years	50	11	43	5	3

### Study Procedures

Medical histories were obtained along with a MoCA for purposes of screening for possible cognitive impairment. Participants then completed a neuroimaging assessment that included acquisition of brain MRI and MRS (see section “Neuroimaging Acquisition and Processing” for imaging sequence details) and cognitive assessment including the National Institutes of Health Toolbox (NIH Toolbox), Trail Making Test Parts A and B, and the California Verbal Learning Test -2 (CVLT-2).

### Cogntive Measures

The MoCA is a 10-min, 30-point clinical assessment of multiple cognitive functions including orientation (6 points), attention (6 points), short-term memory recall (5 points), abstract thinking (2 points), visuospatial executive function assessed by a clock-drawing task, trails task, and reproducing a geometrical figure (5 points), naming task (3 points), and language function assessed by a verbal fluency test (3 points). An additional one point was added for subjects with less than or equal to 12 years in education. People with MoCA scores less than 20 were excluded to decrease the probability of including people with dementia.

The NIH Toolbox includes measures of both crystallized and fluid cognitive abilities. It can be completed in 30 min. It is computerized and well-standardized with norms from a large national cohort of older adults. Discrepancy between crystallized and fluid metrics may be a sensitive metric accounting for premorbid cognitive ability. In most people, early in adulthood, there is not a large discrepancy between crystallized and fluid abilities. In this sample, we have previously published data demonstrating that discrepancy between fluid and crystallized abilities are associated with physical engagement in older adults (O’Shea et al., 2018).

The California Verbal Learning Test −2 is a commonly used supra-span list learning test with five learning trials, an interference list, short delayed free recall, cued recall (by semantic category), long delayed free recall, long delayed cued recall, and a yes-no recognition discrimination task. It can be used to examine different aspects of encoding and rate of forgetting and is sensitive to different processes associated with aging including effects of cerebrovascular disease and Alzheimer’s disease (Libon et al., 1998).

The Trail Making Test Parts A and B is a measure of processing speed and set shifting, often considered to be subsumed within the category of “executive function.” Speed of completion of the trail making test is associated with regional changes in white matter in aging.

### Neuroimaging Acquisition and Processing

All participants were imaged on a 3T Philips Achieva scanner (Philips Healthcare, Best, Netherlands) using a 32-channel head coil at the McKnight Brain Institute (University of Florida, Gainesville, FL, United States). To limit motion during the scan, foam padding was placed in the head coil. A T_1_-weighted anatomical image (magnetization-prepared rapid gradient-echo; repetition time/echo time = 8 ms/3.7 ms, 1-mm 3 isotropic voxels) was acquired for MRS voxel placement and anatomical analysis.

T_1_-weighted MRI scans were processed with the software, FreeSurfer version 5.3. To measure structural volumes, the automated subcortical segmentation stream in FreeSurfer was used. The software uses Bayesian inference methods relying on prior anatomical probabilities in a labeled data set, along with *a priori* known T_1_ intensity characteristics of subcortical regions, as well as T_1_ intensity information from the scan being processed, in order to label discrete regions. Previous research has shown this automated procedure produces accurate and reliable results, while taking a fraction of the time of the gold standard of manual segmentation. This makes automated segmentation well suited for large samples. Any errors in segmentation identified via visual inspection were fixed manually using Freesurfer’s editing tools, and were re-processed through FreeSurfer, as described in Freesurfer’s documentation. This workflow produces results that have been validated against manual segmentation (Morey et al., 2009) and histological measures (Cardinale et al., 2014). Whole volumes were computed as a sum of left and right hemisphere measures; this measure was then normalized in respect to total intracranial volume. All subsequent volumetric indices (e.g., hippocampal volume) refer to this normalized bilateral value. The default anatomical brain atlas in FreeSurfer, Desikan/Killiany atlas, was used. Regions of interest include the hippocampus, parahippocampus, thalamus, amygdala, pallidum, putamen, anterior cingulate, entorhinal cortex, caudate, corpus callosum, subcortical gray matter, cortical gray matter, insula, and cortical white matter.

### MRS Data Acquisition and Processing

**FIGURE 1:**
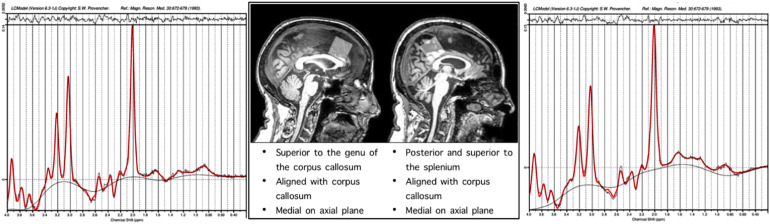
Representative single subject spectra are presented for the frontal and parietal voxels, the *x* axis of the spectra correspond to the chemical shift in PPM, the *y* axis height is proportional to concentration of metabolites.

Single-voxel ^1^H spectra PRESS MRS were collected in frontal and parietal regions. The frontal voxel was collected medially, aligned with the corpus column and superior to the genu. The parietal voxel was collected medially, aligned with the corpus callosum and superior to the splenium. Each voxel was 3 cm × 3 cm × 3 cm (27 cm^3^). An expert rater confirmed appropriate voxel placement based on the landmarks by reconstruction in individual subject anatomical space, after data collection using the GannetCoRegister software. The PRESS sequence had a repetition time (TR) of 2 s and an echo time (TE) 68 ms. This sequence was collected as the interleaved “off” spectrum of a MEGA-PRESS MRS sequence whose results have been published previously (Porges et al., 2017a, b). Specification of this sequence were 320 transients with on-off scans alternating every 2 transients; a 16-step phase cycle (with steps repeated for on and off); 2048 data points acquired at a spectral width of 2 kHz; and variable pulse power and optimized relaxation delays (VAPOR) water suppression. Sixteen transients of water-unsuppressed data were also acquired for quantification using the same acquisition variables. Metabolite concentrations including NAA, MI, Cho (choline + phosphoacetylcholine), Glx (Glu + Gln), and Cr (creatine + phosphocreatine) were determined using the LC Model spectral analysis software and the water unsuppressed acquisition. Voxel locations and example chemical spectra for each are depicted in Figure 1.

### Statistical Analyses

Neuroimaging data were analyzed using a region of interest (ROI) approach whereby volumes of limbic brain regions as well as components of the basal ganglia and cortical and subcortical gray and white matter were analyzed. Proton MRS measures were corrected for the tissue volume within the respective MRS voxel–i.e., [Metabolite]/(1-CSF_Fraction)–and thus are a measure of the concentration within tissue, rather than an absolute estimate of concentration.

Standardized proton MRS indices (NAA, MI, Cho, Glx, and Cr) were entered as predictors into Adaptive Least Absolute Shrinkage and Selection Operator (LASSO) regression models to perform variable selection for predicting each volumetric ROI. These analyses were conducted using the “glmnet” package (Friedman et al., 2010) implemented in R. The adaptive LASSO (Zou, 2006) employs weighted *L*_1_-norm regularization, using a compound cost function to optimize regression coefficients. In this function, λ is a parameter for controlling the strength of *L*_1_-norm regularization. K-fold cross validation was performed using 10 folds to determine the optimal lambda value, which was defined as the one which produced the smallest average mean squared error across folds. In addition, the vector *w* assigns the weight of the *L*_1_ penalty for each coefficient, and *w* was defined in these models as the inverse of the absolute value of the cross-validated ridge regression coefficients. The adaptive LASSO performs variable selection by shrinking the coefficient to zero for any predictors that are not selected in the most parsimonious model for explaining maximal variance in the dependent variable.

After determining the most important predictors for each volumetric ROI from the adaptive LASSO, though technically this is post-selection inference, we performed multiple linear regressions using the selected variables as predictors for each ROI (see Table 3). Since the adaptive LASSO has the “oracle property” this has theoretical justification and is commonly paired with linear regression to further extrapolate findings. Age was entered as a predictor in a separate adaptive LASSO model with the volumetric ROIs and the regional metabolites to determine how age factors into the selection models and post-inferential linear regression models. As age may be a proxy for some of the physiological processes captured by changes in metabolic and structural brain volumes, both models are presented (see Table 4). Age was also correlated with the identified regional metabolites and the volumes of each ROI to determine the direction of aging effect independently on these variables. Sex was used as an independent variable in a one-way ANOVA to examine the relationship to the volumes of interest and metabolites of interest. Similar adaptive LASSO regressions and subsequent linear regressions were conducted on the NIH Toolbox crystallized-fluid discrepancy (adjusted by age) and Trail Making Tests Part A and B speed (adjusted by age and sex) using ROI metabolites identified to be relevant to volumetric change and two regions known to be important in cognitive performance in aging, hippocampal and white matter volumes. Likewise, this method was used for MoCA and CVLT-2 recognition discrimination, except in these metrics, as age is not accounted for in the raw values, age was entered as a co-variate. Cognitive, ROI volume, and metabolite descriptive statistics are presented in Table 2.

**TABLE 2:** Descriptive statistics for metabolite concentrations, ROI volumes, and scores on cognitive measures.

	N	Mean	SD
**Metabolite concentrations**			
Frontal Glx	117	3.95	0.72
Frontal MI	117	2.94	0.44
Frontal NAA	117	6.53	0.50
Frontal Cho	117	1.18	0.18
Frontal Cr	117	4.60	0.52
Parietal Glx	117	3.80	0.49
Parietal MI	117	3.29	0.47
Parietal NAA	117	7.04	0.54
Parietal Cho	117	1.13	0.17
Parietal Cr	117	5.06	0.44
**ROI volumes**			
Hippocampus	117	5.31 × 10^–3^	1.04 × 10^–3^
Parahippocampus	117	2.76 × 10^–3^	5.37 × 10^–4^
Insula	117	9.40 × 10^–3^	1.53 × 10^–3^
Anterior cingulate	117	5.20 × 10^–3^	1.03 × 10^–3^
Entorhinal cortex	117	2.77 × 10^–3^	5.04 × 10^–4^
Amygdala	117	2.19 × 10^–3^	4.45 × 10^–4^
Corpus callosum	117	2.40 × 10^–3^	5.06 × 10^–4^
Caudate	117	4.66 × 10^–3^	8.47 × 10^–4^
Thalamus	117	8.94 × 10^–3^	1.50 × 10^–3^
Pallidum	117	2.61 × 10^–3^	5.06 × 10^–4^
Putamen	117	6.06 × 10^–3^	1.21 × 10^–3^
Cortical gray matter	117	0.29	0.04
Subcortical gray matter	117	0.04	0.01
Cortical white matter	117	0.30	0.05
**Cognitive measures**			
MoCA	79	25.76 (raw, out of 30)	2.59
CVLT-2 recognition discrimination	79	7.24 (raw)	1.96
Trails A	79	53.19 (raw time)	8.35
Trails B	79	50.13 (raw time)	9.95
NIH toolbox crystallized – fluid intelligence discrepancy	79	15.11	16.02

*ROI volumes are adjusted by ICV and the resulting value is thus ROI ml^3/^ICV ml^3^.*

**TABLE 3:** Relationships between regional brain volumes and cerebral metabolites.

Frontal voxel							Parietal voxel						
ROI	β	SE	Multiple *R*^2^	Adjusted *R*^2^	*F*	*p*	ROI	β	SE	Multiple *R*^2^	Adjusted *R*^2^	*F*	*p*
***Cortical regions***							***Cortical regions***						
Parahippocampus			0.09	0.07	*F*(3, 113) = 3.79	0.01	Parahippocampus			0.05	0.04	*F*(1, 115) = 5.44	0.02
Glx	−6.84 × 10^–5^	5.61 × 10^–5^				0.23	Cho	−1.14 × 10^–4^	4.89 × 10^–5^				0.02
NAA	2.14 × 10^–4^	6.49 × 10^–5^				1.31 × 10^–3^							
Cr	−1.34 × 10^–3^	5.90 × 10^–5^				0.03							
Insula						NS	Insula			0.13	0.11	*F*(3, 113) = 5.59	1.30 × 10^–3^
							NAA	−1.23 × 10^–4^	1.77 × 10^–4^				0.49
							Cho	−3.58 × 10^–4^	1.52 × 10^–4^				0.02
							Cr	−1.91 × 10^–4^	1.83 × 10^–4^				0.30
Anterior cingulate						NS	Anterior cingulate			0.07	0.07	*F*(1, 115) = 9.14	3.09 × 10^–3^
							Cho	−2.79 × 10^–4^	9.23 × 10^–5^				3.09 × 10^–3^
Entorhinal cortex						NS	Entorhinal cortex			0.06	0.38	*F*(2, 114) = 3.32	0.04
							Glx	8.13 × 10^–5^	4.78 × 10^–5^				0.09
							Cho	−1.12 × 10^–4^	4.78 × 10^–5^				0.02
***Subcortical regions***							***Subcortical regions***						
Amygdala						NS	Amygdala			0.05	0.04	*F*(1, 115) = 5.49	0.02
							Cho	−9.51 × 10^–5^	4.06 × 10^–5^				0.02
Corpus callosum			0.10	0.09	*F*(2, 114) = 6.64	1.88 × 10^–3^	Corpus callosum						NS
NAA	1.55 × 10^–4^	5.49 × 10^–5^				5.65 × 10^–3^							
Cr	−1.93 × 10^–3^	5.49 × 10^–5^				6.42 × 10^–4^							
Caudate			0.08	0.07	*F*(2, 114) = 5.27	6.47 × 10^–3^	Caudate			0.05	0.04	*F*(1, 115) = 5.93	0.02
NAA	2.59 × 10^–3^	9.29 × 10^–5^				6.22 × 10^–3^	Cr	−1.88 × 10^–5^	7.70 × 10^–5^				0.02
Cr	−2.76 × 10^–3^	9.29 × 10^–5^				3.66 × 10^–3^							
Hippocampus			0.07	0.05	*F*(2, 114) = 4.26	0.02	Hippocampus			0.06	0.05	*F*(1, 115) = 7.55	6.96 × 10^–3^
NAA	3.04 × 10^–4^	1.15 × 10^–4^				9.63 × 10^–3^	Cho	−2.59 × 10^–4^	9.43 × 10^–5^				6.96 × 10^–3^
Cr	−2.94 × 10^–4^	1.15 × 10^–4^				0.01							
Thalamus			0.08	0.07	*F*(4, 112) = 3.04	6.99 × 10^–3^	Thalamus			0.08	0.07	*F*(1, 115) = 9.94	2.07 × 10^–3^
NAA	4.75 × 10^–4^	1.65 × 10^–4^				4.78 × 10^–3^	Cho	−4.24 × 10^–4^	1.34 × 10^–4^				2.07 × 10^–3^
Cr	−4.69 × 10^–4^	1.65 × 10^–4^				5.33 × 10^–3^							
Pallidum						NS	Pallidum			0.09	0.08	*F*(1, 115) = 10.72	1.40 × 10^–3^
							Cho	−1.48 × 10^–4^	4.52 × 10^–5^				1.40 × 10^–3^
Putamen			0.12	0.09	*F*(4, 112) = 3.81	6.06 × 10^–3^	Putamen			0.06	0.05	*F*(2, 114) = 3.82	0.02
Glx	−2.02 × 10^–4^	1.26 × 10^–4^				0.11	Cho	−1.62 × 10^–4^	1.22 × 10^–3^				0.19
NAA	5.15 × 10^–4^	1.45 × 10^–4^				5.40 × 10^–4^	Cr	−1.94 × 10^–4^	1.22 × 10^–3^				0.12
Cho	1.33 × 10^–4^	1.40 × 10^–4^				0.34							
Cr	−4.35 × 10^–4^	1.53 × 10^–4^				5.38 × 10^–3^							
***Global measures***							***Global measures***						
Cortical gray matter			0.10	0.08	*F*(2, 114) = 6.14	2.93 × 10^–3^	Cortical gray matter			0.09	0.08	*F*(1, 115) = 10.94	1.25 × 10^–3^
NAA	0.02	4.78 × 10^–3^				1.90 × 10^–3^	Cho	−0.01	3.92 × 10^–3^				1.25 × 10^–3^
Cr	−0.01	4.78 × 10^–3^				2.95 × 10^–3^							
Subcortical gray matter			0.10	0.08	*F*(3, 113) = 4.42	5.63 × 10^–3^	Subcortical gray matter			0.08	0.06	*F*(2, 114) = 4.83	9.71 × 10^–3^
Glx	−8.81 × 10^–4^	6.23 × 10^–4^				0.16	Cho	−1.33 × 10^–3^	6.03 × 10^–4^				0.03
NAA	2.41 × 10^–3^	7.30 × 10^–4^				1.10 × 10^–3^	Cr	−5.93 × 10^–4^	6.03 × 10^–4^				0.33
Cr	−1.86 × 10^–3^	6.55 × 10^–4^				5.32 × 10^–3^							
Cortical white matter			0.10	0.09	*F*(2, 114) = 6.40	2.33 × 10^–3^	Cortical white matter			0.04	0.03	*F*(1, 115) = 4.93	0.03
NAA	0.02	4.98 × 10^–3^				1.20 × 10^–3^	Cho	−9.28 × 10^–3^	4.18 × 10^–3^				0.03
Cr	−0.01	4.98 × 10^–3^				3.32 × 10^–3^							

*Variables reported were selected by the adaptive LASSO models as important predictors of regional volumes. Absolute metabolite measures are included. Volumetric measures have been adjusted by total intracranial volume. Adjusted R^2^ values are for the whole model fit. Note: NS indicates that none of the metabolites were selected by the adaptive LASSO.*

**TABLE 4:** Relationships between regional brain volumes and metabolites with age in the model.

Frontal voxel							Parietal voxel						
ROI	β	SE	Multiple *R*^2^	Adjusted *R*^2^	*F*	*p*	ROI	β	SE	Multiple *R*^2^	Adjusted *R*^2^	*F*	*p*
***Cortical regions***							***Cortical regions***						
Parahippocampus			0.17	0.14	*F*(3, 113) = 7.57	1.17 × 10^–4^	Parahippocampus			0.15	0.14	*F*(2, 114) = 10.22	2.51 × 10^–8^
NAA	1.35 × 10^–4^	5.80 × 10^–5^				0.02	Cho	−8.97 × 10^–5^	4.67 × 10^–5^				0.06
Cr	−1.07 × 10^–4^	5.71 × 10^–5^				0.06	Age	−1.77 × 10^–4^	4.67 × 10^–5^				2.41 × 10^–4^
Age	−1.64 × 10^–4^	4.74 × 10^–5^				7.78 × 10^–4^							
Insula			0.07	0.06	*F*(1, 115) = 9.00	3.32 × 10^–3^	Insula			0.18	0.15	*F*(4, 112) = 6.10	1.78 × 10^–4^
Age	−4.13 × 10^–4^	1.38 × 10^–4^				3.32 × 10^–3^	NAA	−1.31 × 10^–4^	1.73 × 10^–4^				0.45
							Cho	−3.16 × 10^–4^	1.49 × 10^–4^				0.04
							Cr	−1.71 × 10^–4^	1.79 × 10^–4^				0.34
							Age	−3.45 × 10^–4^	1.33 × 10^–4^				0.01
Anterior cingulate			0.11	0.11	*F*(1, 115) = 14.93	1.85 × 10^–4^	Anterior cingulate			0.17	0.15	*F*(2, 114) = 11.37	3.14 × 10^–5^
Age	−3.49 × 10^–4^	9.02 × 10^–5^				1.85 × 10^–4^	Cho	−2.35 × 10^–4^	8.88 × 10^–5^				9.16 × 10^–3^
							Age	−3.16 × 10^–4^	8.88 × 10^–5^				5.42 × 10^–4^
Entorhinal cortex			0.09	0.06	*F*(1, 115) = 2.78	0.01	Entorhinal cortex			0.10	0.07	*F*(3, 113) = 3.97	9.85 × 10^–3^
Age	−1.14 × 10^–4^	4.58 × 10^–5^				0.01	Glx	7.88 × 10^–5^	4.70 × 10^–5^				0.10
							Cho	−9.69 × 10^–5^	4.75 × 10^–5^				0.04
							Age	−1.02 × 10^–4^	4.56 × 10^–5^				0.02
***Subcortical regions***							***Subcortical regions***						
Amygdala			0.29	0.28	*F*(1, 115) = 46.28	4.87 × 10^–10^	Amygdala			0.31	0.29	*F*(2, 114) = 25.23	8.50 × 10^–10^
Age	−2.39 × 10^–4^	3.51 × 10^–5^				4.87 × 10^–10^	Cho	−6.34 × 10^–5^	3.51 × 10^–5^				0.07
							Age	−2.30 × 10^–4^	3.51 × 10^–5^				1.69 × 10^–9^
Corpus callosum			0.24	0.22	*F*(3, 113) = 11.96	7.39 × 10^–7^	Corpus callosum			0.18	0.17	*F*(1, 115) = 24.95	2.12 × 10^–6^
NAA	1.01 × 10^–4^	5.22 × 10^–5^				0.06	Age	−2.14 × 10^–4^	4.28 × 10^–5^				2.12 × 10^–6^
Cr	−1.57 × 10^–4^	5.14 × 10^–5^				2.85 × 10^–3^							
Age	−1.92 × 10^–4^	4.26 × 10^–5^				1.59 × 10^–5^							
Caudate			0.11	0.09	*F*(3, 113) = 4.68	4.03 × 10^–3^	Caudate			0.09	0.07	*F*(2, 114) = 5.50	5.25 × 10^–3^
NAA	2.20 × 10^–4^	9.45 × 10^–5^				0.02	Cr	−1.71 × 10^–4^	7.61 × 10^–5^				0.03
Cr	−2.49 × 10^–4^	9.31 × 10^–5^				8.48 × 10^–3^	Age	−1.68 × 10^–4^	7.61 × 10^–5^				0.03
Age	−1.40 × 10^–4^	7.72 × 10^–5^				0.07							
Hippocampus			0.23	0.22	*F*(1, 115) = 34.47	4.28 × 10^–8^	Hippocampus			0.26	0.25	*F*(2, 114) = 20.49	2.51 × 10^–8^
Age	−5.01 × 10^–4^	8.54 × 10^–5^				4.28 × 10^–8^	Cho	−1.94 × 10^–4^	8.46 × 10^–5^				0.02
							Age	−4.74 × 10^–4^	8.46 × 10^–5^				1.47 × 10^–7^
Thalamus			0.28	0.27	*F*(1, 115) = 44.38	9.76 × 10^–10^	Thalamus			0.32	0.31	*F*(2, 114) = 27.21	2.19 × 10^–10^
Age	−7.93 × 10^–4^	1.19 × 10^–4^				9.76 × 10^–10^	Cho	−3.21 × 10^–4^	1.17 × 10^–4^				7.09 × 10^–3^
							Age	−7.49 × 10^–4^	1.17 × 10^–4^				3.49 × 10^–9^
Pallidum			0.11	0.10	*F*(1, 115) = 13.72	3.27 × 10^–4^	Pallidum			0.17	0.15	*F*(2, 114) = 11.58	2.65 × 10^–5^
Age	−1.65 × 10^–4^	4.46 × 10^–5^				3.27 × 10^–4^	Cho	−1.28 × 10^–4^	4.37 × 10^–5^				4.21 × 10^–3^
							Age	−1.48 × 10^–4^	4.37 × 10^–5^				9.78 × 10^–4^
Putamen			0.22	0.19	*F*(4, 112) = 7.94	1.14 × 10^–5^	Putamen			0.20	0.18	*F*(3, 113) = 9.33	1.47 × 10^–5^
Glx	−1.51 × 10^–4^	1.18 × 10^–4^				0.20	Cho	−1.09 × 10^–4^	1.14 × 10^–4^				0.34
NAA	3.89 × 10^–4^	1.40 × 10^–4^				6.36 × 10^–3^	Cr	−1.74 × 10^–4^	1.14 × 10^–4^				0.13
Cr	−2.85 × 10^–4^	1.25 × 10^–4^				0.02	Age	−4.50 × 10^–4^	1.03 × 10^–4^				2.73 × 10^–5^
Age	−4.10 × 10^–4^	1.04 × 10^–4^				1.41 × 10^–4^							
***Global measures***							***Global measures***						
Cortical gray matter			0.27	0.25	*F*(4, 112) = 10.53	2.80 × 10^–7^	Cortical gray matter			0.27	0.26	*F*(2, 114) = 21.48	1.22 × 10^–8^
NAA	0.01	4.48 × 10^–3^				0.02	Cho	−0.01	3.54 × 10^–3^				4.33 × 10^–3^
Cho	−4.65 × 10^–3^	4.59 × 10^–3^				0.31	Age	−0.02	3.54 × 10^–3^				3.46 × 10^–7^
Cr	−8.40 × 10^–3^	5.12 × 10^–3^				0.10							
Age	−0.02	3.64 × 10^–3^				1.19 × 10^–6^							
Subcortical gray matter			0.25	0.23	*F*(3, 113) = 12.74	3.11 × 10^–7^	Subcortical gray matter			0.25	0.24	*F*(2, 114) = 19.21	6.68 × 10^–7^
NAA	1.28 × 10^–3^	6.14 × 10^–4^				0.04	Cho	−1.23 × 10^–3^	4.91 × 10^–4^				0.01
Cr	−1.44 × 10^–3^	6.05 × 10^–4^				0.02	Age	−2.58 × 10^–3^	4.91 × 10^–4^				26.68 × 10^–7^
Age	−2.50 × 10^–3^	5.02 × 10^–4^				2.34 × 10^–6^							
Cortical white matter			0.36	0.34	*F*(3, 113) = 21.27	5.38 × 10^–11^	Cortical white matter			0.34	0.33	*F*(2, 114) = 28.99	6.64 × 10^–11^
NAA	9.79 × 10^–3^	4.33 × 10^–3^				0.03	Cho	−5.82 × 10^–3^	3.52 × 10^–3^				0.10
Cr	−0.01	4.27 × 10^–3^				0.02	Age	−0.03	3.52 × 10^–3^				9.58 × 10^–11^
Age	−0.02	3.54 × 10^–3^				5.74 × 10^–10^							

*Variables reported were selected by the adaptive LASSO as important predictors of regional volumes. Absolute metabolite measures are included. Volumetric measures have been adjusted by total intracranial volume. Adjusted R^2^ values are for the whole model fit.*

## Results

The MRS indices along with covariates selected by the adaptive LASSO regression for specific brain ROI volumes and the results from the subsequent multiple linear regression models are indicated in Tables 3, 4 (with age as a selectable variable). Cognitive results are presented in Table 6.

### Frontal Metabolites

Adaptive LASSO selected NAA and Cr as predictors of hippocampal, cortical gray matter, cortical white matter, corpus callossum, caudate, and thalamic volumes. Further, NAA, Glx, and Cr were selected as predictors of parahippocampal and subcortical gray matter volume. Finally, Cho was an additional predictor to NAA, Glx, and Cr of Putamen volumes. Across all of the above regions, NAA was a significant predictor in post-inferential linear regressions.

With age as a selectable variable in the adaptive LASSO, age is the only identified predictor of hippocampal, amygdala, insula, anterior cingulate, entorhinal cortex, thalamic, and pallidum volumes.

### Parietal Metabolites

Adaptive LASSO selected Cho as the predictor of hippocampus, parahippocampal, amygdala, anterior cingulate, cortical gray matter, cortical white matter, caudate, thalamic, and pallidum volumes. Further, Cho and Cr were selected as important in subcortical gray matter and putamen volumes. NAA, Cho, and Cr were important in insular volume. Across all of the above regions, Cho was a significant predictor in post-inferential linear regression models.

With age as a selectable variable in the adaptive LASSO, age was the only predictor in one model, corpus callosum, and was identified as an important variable in all models except the pallidum.

### Age and Sex

Age was not statistically significantly associated with either frontal lobe NAA or parietal lobe Cho, but the direction was such that as Cho was positively associated (*r* = 0.138) with age and NAA negatively associated (*r* = −0.173). Age was negatively associated with all ROI volumes (hippocampus −0.0.480, parahippocampus −0.399, thalamus −0.528, amygdala −0.536, pallidum −0.327, putamen −0.399, anterior cingulate −0.339, entorhinal cortex −0.227, caudate −0.218, corpus callosum −0.422, subcortical gray matter −0.459, cortical gray matter −0.469, insula −0.269, and cortical white matter −0.567). Generally, frontal NAA and parietal Cho remained as important predictors of ROI volumes with age as a selectable variable in the adaptive LASSO analysis. Those results are summarized in Table 4.

We analyzed the effects of sex on the relationships of regional metabolites to structural volumes. Men versus women did not statistically differ as a function of age- both had a mean age of 71 years. Sex is significantly associated with all regional volumes (generally women had higher volumes). Further, sex is significantly associated with frontal lobe Cho and Cr (women lower). Sex is also associated with parietal MI and Cho (women lower). Within sex, the direction of relationship between frontal lobe NAA and regional volumes is the same. Further, the direction of relationship between parietal Cho and regional volumes is the same with the exception of caudate volume in which Cho is positively, though not significantly, correlated in men (*r* = 0.243, *p* = 0.097) and negatively, though not significantly, correlated, in women (*r* = -0.229, *p* = 0.059). In Adaptive LASSO models including sex as a selectable variable, the pattern of results is consistent with our other analyses. For frontal metabolites, NAA was the most commonly selected predictor of structural volumes, and for parietal metabolites, Cho was the most commonly selected predictor of structural volumes. Along with sex, NAA was selected for: parahippocampus, cortical gray matter, cortical white matter, corpus callosum, caudate, thalamus, and putamen volumes. For parietal metabolites, along with sex, Cho is still selected for: parahippocampus, insula, ACC, cortical gray matter, subcortical gray matter, thalamus, and pallidum. Results of LASSO analyses within sex are presented in Table 5.

**TABLE 5:** LASSO regression selected indicators by Sex.

ROI	Frontal voxel		Parietal voxel	
	
	Women	Men	Women	Men
*Cortical regions*				
Parahippocampus	NAA	Glx NAA Cho		
Insula		Glx Cr	NAA Cho	
Anterior cingulate	NAA		Glx NAA Cho	
Entorhinal cortex				Glx MI Cho
*Subcortical regions*				
Amygdala			Cho	
Corpus callosum	NAA Cho Cr			
Caudate		NAA Cho Cr	Cr	MI Cho
Hippocampus	NAA		NAA Cho	
Thalamus	NAA Cr		Cho	Cho
Pallidum		NAA	Cho	
Putamen		NAA Cho Cr	Cho Cr	
*Global measures*				
Cortical gray matter	NAA		Cho	MI Cho
Subcortical gray matter	NAA		Cho	
Cortical white matter	NAA Cr		NAA	Cho

### Cognitive Performance

Adaptive LASSO selected hippocampal volume as a predictor of MoCA total score. Further, hippocampal volume, cortical white matter, and age were selected as predictors of CVLT-2 recognition discrimination, and parietal Cho and hippocampal volume were selected as predictors of the NIH Toolbox crystallized-fluid intelligence discrepancy. It should be noted that the direction of this relationship was such that Cho was negatively associated with the discrepancy. For Trail Making Tests Part A and B, using age, sex and race corrected norms, none of the predictors were selected by the adaptive LASSO. Though we did not have aprior predictions, we ran an additional LASSO analysis on CVLT-2 metrics (total learning, trials 1 through 4), short delayed free recall, and long delay free recall. The adaptive LASSO selected cortical white matter as a predictor of total learning, cortical white matter, frontal NAA, parietal Cho, and hippocampal volume as important predictors of short delayed free recall and parietal Cho and hippocampal volume as important predictors of long delayed free recall. In post-inferential linear regression models, none of these predictors were statistically significant. Results are summarized in Table 6. Note, the cognitive measures were administered in a subset of the sample due to technical limitations at the start of the study. There was no systematic difference in participants who received cognitive testing compared to those who did not.

**TABLE 6:** Relationships between frontal NAA, parietal Cho, cortical white matter volume, and hippocampal volume to cognitive performance.

ROI	β	SE	Multiple *R*^2^	Adjusted *R*^2^	*F*	*P*
MoCA			0.10	0.09	*F*(1, 77) = 8.57	4.50 × 10^–3^
Hippocampus	0.84	0.29				4.50 × 10^–3^
CVLT discrimination			0.17	0.14	*F*(3, 75) = 5.10	2.89 × 10^–3^
Cortical white matter	−1.17	0.46				0.01
Hippocampus	1.16	0.41				5.54 × 10^–3^
Age	−0.55	0.26				0.04
Trails A						NS
Trails B						NS
Crystallized – fluid discrepancy			0.12	0.10	*F*(2, 76) = 5.20	7.66 × 10^–3^
Parietal Cho	−3.69	1.74				0.04
Hippocampus	−5.25	1.82				5.05 × 10^–3^

*Variables reported were selected by the adaptive LASSO models as important predictors of regional volumes. p-values are generated via post-inferential linear regression. Adjusted R^2^ values are for the whole model fit.*

## Discussion

Prefrontal and parietal cerebral metabolite concentrations were associated with structural brain volumes in older adults who were not exhibiting declines in cognitive abilities or functional status indicative of MCI or a neurogenerative disorder. Greater cerebral NAA concentrations in the prefrontal cortex corresponded with greater volumes of the parahippocampus, hippocampus, caudate, thalamus, and putamen as well as whole brain cortical and subcortical gray matter, cortical white matter, and corpus callosum. In contrast, greater Cho concentrations in the parietal cortex corresponded with reduced hippocampal, parahippocampal, amygdala, insular, anterior cingulate, thalamic, pallidum, and putamen volumes as well as whole brain cortical and subcortical gray matter and cortical white matter volumes. These results extend past findings linking cerebral metabolite concentrations with the volumes of cortical and subcortical areas in patients with specific pathologies including HIV and congenital heart disease (Cohen et al., 2010; Gertsvolf et al., 2018), as well as evidence demonstrating neurochemical changes in both normal and pathological aging (Ding et al., 2016). In the context of these age-associated pathologies, elevated or reduced regional concentrations of specific cerebral metabolites have been linked to pathophyisiological abnormalities, including neuronal damage, cell membrane breakdown, inflammatory pathophysiology, and neurotoxicity (Voevodskaya et al., 2019). Evidence of associations between cerebral metabolite concentrations and cortical and subcortical volumes in the current study is noteworthy given that the cohort consisted of older adults without MCI, AD, or other known neurological brain disorders. The results suggest that relationships between concentration of cerebral metabolites and volumes exist in older adults even in the context of normal cognitive aging, and may reflect age-associated metabolic and neurophysiological alterations that affect the structure of the brain.

It is noteworthy that the observed metabolite-volumetric relationships occurred in regions that were not confined to where MRS was sampled. The cerebral metabolites that were associated with the volumes of specific cortical and subcortical volumes differed according to whether the metabolites were measured in the frontal or parietal cortex, suggesting different age-associated metabolic and neurophysiological alterations occurring in the frontal and parietal cortices. These results also suggest that measurement of metabolites performed in a specific cortical region may be sensitive to changes occurring in connected regions, possibly reflecting interrelated pathophysiological processes. Though NAA in prefrontal cortex was most consistently related to limbic/subcortical brain volumes, Cho was most salient in parietal cortex. This finding is potentially significant given the role that inflammatory processes are known to play in biological aging (Franceschi et al., 2007). Not surprisingly, age is an important predictor of all structural volumes tested. It is noteworthy, that with age as a selectable variable, that frontal lobe NAA and parietal lobe Cho remain important predictors of structural volumes. In post inferential linear regression analyses, we see that age accounts for some of the variance in the relationships of these metabolites to structural volumes, suggesting that, although age is not significantly related to either metabolite level, that the aging process, or correlates therein, is playing a role in these associations. Further, the direction of the relationship between frontal lobe NAA and parietal lobe Cho to structural volumes is the same within both men and women. Individual differences in chronic stress, length of stress, and genetic risk factors as well as differences in exercise and dietary choices may have a substantial impact on brain aging (Woods et al., 2013; Seider et al., 2016). The association between metabolic concentrations and brain structures specific to NAA in prefrontal cortex but not parietal cortex is consistent with other structural and functional imaging work in normal aging. Cho in parietal cortex may be indicative of inflammation associated with vascular changes, or perhaps a diaschisis effect suggesting an emergent process.

These metabolite concentrations may be important in the context of age-associated volume losses, previously reported, in medial temporal lobe (MTL; Jack et al., 1998). Further, in functional connectivity studies of normal aging, there may be disruption of functional connectivity in areas including medial frontal gyrus and dorsolateral prefrontal cortex (Pagani et al., 2017). Common diseases associated with aging including hypertension, diabetes and other cerebrovascular risk factors may preferentially affect fronto-subcortical function. This may include pre-clinical manifestations of these systemic inefficiencies and pathologies. White matter hyper intensities anywhere in the brain are associated with hypometabolism in prefrontal cortex (Tullberg et al., 2004).

The differential susceptibility of regions of the brain to volume loss and their relationship to cerebral metabolite levels measured by proton MRS points to the potential power of these methods for studying and measuring the effects of brain aging factors. The fact that either elevated or reduced concentrations of these cerebral metabolites are associated with brain disorders raises the possibility that abnormal levels of these cerebral metabolites may be leading indicators of underlying patterns of structural decay resulting in future cognitive decline. Different MRS metabolites in frontal cortex and parietal cortex were strong predictors of brain structural changes elsewhere in cognitively salient structures including the hippocampus, white matter, thalamus, and basal ganglia. These include brain indicators of possible neuroinflammation. Likewise, peripheral inflammatory factors have been shown by others to be associated with adiposity including interleukin (IL) −6 and C-reactive protein (CRP) in midlife adults are associated with changes in cognition and hippocampal volumes (Marsland et al., 2015).

Of note, parietal Cho and hippocampal volume were both associated with the amount of discrepancy between the crystallized and fluid cognition function indices of the NIH-TB, though the Cho concentration is not in the expected direction (negative association). Among adults without neurological brain disturbances affecting specific cognitive domains, for example stroke affecting language, crystalized cognitive functions (e.g., vocabulary knowledge) are usually very stable across the lifespan. Crystalized cognitive performance therefore tends to reflect pre-morbid abilities, or in the context of aging, how an individual functioned when they were younger. In contrast, fluid cognitive functions (e.g., working memory, processing speed, etc.) are more susceptible to various conditions that affect the brain, and also performance in these cognitive domains declines with advanced age. Consequently, age-adjusted norms are essential for determining how an individual is performing relative to expectations for people of a similar age. We have previously shown that the discrepancy between crystalized and fluid cognitive performance is associated with social and physical activity (O’Shea et al., 2018).

In the present study, older adults with elevated Cho concentrations in the parietal cortex tended to have a greater discrepancy score, with fluid cognitive performance weaker than crystalized abilities. This finding suggests that even though the study participants were “cognitively healthy” by clinical standards, those with weaker fluid relative to crystalized cognitive performance were exhibiting cerebral metabolic abnormalities in the parietal cortex. While neuroinflammation may be one factor contributing to age-associated brain changes, reduced neural integrity may be caused by other factors that are not tied to inflammation.

This study has limitations. It should be emphasized that these findings are based on cross-sectional analyses. What we have reported are associations, and causality as well as potential pathological association including vascular risk factors, needs to be assessed via other methods. It is important to note that participants in this sample included people with common chronic conditions in aging such as hypertension and type 2 diabetes. Further, sample size is a limiting factor as is potential selection bias and potential unmeasured confounders. Also, sample size limits interpretation of in depth analyses of sex differences and interactions in relationships of metabolites to ROI volumes.

## Conclusion

Overall, multiple metabolically relevant associations in prefrontal cortex as indicated by NAA and parietal cortex as indicated by Cho concentration variances are associated with different aspects of regional brain changes in this cross-sectional study. These may be indicators of later functional decline including mild cognitive impairment or early manifestation of Alzheimer’s disease changes (hippocampal volume and forgetting). In our sample, parietal Cho, hippocampal volume, and fluid/crystallized cognition discrepancy were associated, and hippocampal volume was significantly associated with relative forgetting in our apparently cognitively healthy sample. Further, associations may potentially reflect cerebrovascular disease process, given relationships between NAA and white matter volume in our sample. The current data illustrate a dissociation between frontal and parietal metabolites and relationship to regional differences in cortical and subcortical structures, demonstrating sensitivity of these potentially pathophysiologically associated metabolites and structures known to underlie specific cognitive changes in aging. The current data also are suggestive of potentially pathophysiological differentiation in apparently cognitively healthy agers that need to be studied longitudinally to assess progressive differences in outcomes.

## Data Availability Statement

The datasets presented in this article are not readily available because only a limited dataset may be available, per University of Florida IRB and funding (MBRF) rules. Requests to access the datasets should be directed to roncohen@ufl.edu.

## Ethics Statement

The studies involving human participants were reviewed and approved by University of Florida, Institutional Review Board. The patients/participants provided their written informed consent to participate in this study.

## Author Contributions

JW contributed to the design of the study, analyzed the data, composed elements of the statistics section and table, wrote the initial draft of a manuscript, integrated edits from other authors, and responsible for edits and final draft of the manuscript. DL performed the statistical analysis on a manuscript, wrote the initial draft of the stats and analysis section, discussed the interpretation of data, and addressed imaging methodological components. EP wrote MRS methodology, contributed to the design of the study, and discussed interpretation of data. SB performed the statistical analysis, drafted the tables, contributed to the interpretation of results, and wrote the elements of the discussion. AW contributed to design of the study, conceptualization of manuscript, editing, and interpretation of data. SD determined statistical approach, contributed to the interpretation of data, and edited the presentation of results. KL contributed to literature review and writing, and framing cognitive components of the study. RC contributed to design of the study, analyzed the data, wrote the elements of discussion, and edited the manuscript. All authors contributed to the article and approved the submitted version.

## Disclaimer

The views expressed in this article are those of the authors and do not necessarily reflect the position or policy of the Department of Veterans Affairs or the United States government.

## Conflict of Interest

The authors declare that the research was conducted in the absence of any commercial or financial relationships that could be construed as a potential conflict of interest.

## References

[B1] Ahluwalia V. Wade J. B. Thacker L. Kraft K. A. Sterling R. K. Stravitz R. T. (2013). Differential impact of hyponatremia and hepatic encephalopathy on health-related quality of life and brain metabolite abnormalities in cirrhosis. *J. Hepatol.* 59 467–473. 10.1016/j.jhep.2013.04.023 23665182 PMC3748234

[B2] Berridge M. J. Irvine R. F. (1989). Inositol phosphates and cell signalling. *Nature* 341 197–205. 10.1038/341197a0 2550825

[B3] Burger A. Brooks S. J. Stein D. J. Howells F. M. (2018). The impact of acute and short-term methamphetamine abstinence on brain metabolites: a proton magnetic resonance spectroscopy chemical shift imaging study. *Drug Alcohol. Depend.* 185 226–237. 10.1016/j.drugalcdep.2017.11.029 29471227

[B4] Cardinale F. Chinnici G. Bramerio M. Mai R. Sartori I. Cossu M. (2014). Validation of FreeSurfer-estimated brain cortical thickness: comparison with histologic measurements. *Neuroinformatics* 12 535–542. 10.1007/s12021-014-9229-2 24789776

[B5] Cohen R. A. Harezlak J. Gongvatana A. Buchthal S. Schifitto G. Clark U. (2010). Cerebral metabolite abnormalities in human immunodeficiency virus are associated with cortical and subcortical volumes. *J. Neurovirol.* 16 435–444. 10.1007/bf03210849 20961212 PMC4560459

[B6] Crivello F. Tzourio-Mazoyer N. Tzourio C. Mazoyer B. (2014). Longitudinal assessment of global and regional rate of grey matter atrophy in 1,172 healthy older adults: modulation by sex and age. *PLoS One* 9:e114478. 10.1371/journal.pone.0114478 25469789 PMC4255026

[B7] Cysique L. A. Juge L. Gates T. Tobia M. Moffat K. Brew B. J. (2018). Covertly active and progressing neurochemical abnormalities in suppressed HIV infection. *Neurol. Neuroimmunol. Neuroinflamm.* 5:e430. 10.1212/nxi.0000000000000430 29312999 PMC5754644

[B8] Ding X. Q. Maudsley A. A. Sabati M. Sheriff S. Schmitz B. Schütze M. (2016). Physiological neuronal decline in healthy aging human brain–an in vivo study with MRI and short echo-time whole-brain (1)H MR spectroscopic imaging. *Neuroimage* 137 45–51. 10.1016/j.neuroimage.2016.05.014 27164326 PMC4914466

[B9] Driscoll I. Davatzikos C. An Y. Wu X. Shen D. Kraut M. (2009). Longitudinal pattern of regional brain volume change differentiates normal aging from MCI. *Neurology* 72 1906–1913. 10.1212/wnl.0b013e3181a82634 19487648 PMC2690968

[B10] Fletcher E. Gavett B. Harvey D. Farias S. T. Olichney J. Beckett L. (2018). Brain volume change and cognitive trajectories in aging. *Neuropsychology* 32 436–449. 10.1037/neu0000447 29494196 PMC6525569

[B11] Foong J. Rozewicz L. Davie C. A. Thompson A. J. Miller D. H. Ron M. A. (1999). Correlates of executive function in multiple sclerosis: the use of magnetic resonance spectroscopy as an index of focal pathology. *J. Neuropsychiatry Clin. Neurosci.* 11 45–50. 10.1176/jnp.11.1.45 9990555

[B12] Franceschi C. Capri M. Monti D. Giunta S. Olivieri F. Sevini F. (2007). Inflammaging and anti-inflammaging: a systemic perspective on aging and longevity emerged from studies in humans. *Mech. Ageing Dev.* 128 92–105. 10.1016/j.mad.2006.11.016 17116321

[B13] Friedman J. Hastie T. Tibshirani R. (2010). Regularization paths for generalized linear models via coordinate descent. *J. Stat. Softw.* 33:1. PMC2929880 20808728

[B14] Gertsvolf N. Votava-Smith J. K. Ceschin R. del Castillo S. Lee V. Lai H. A. (2018). Association between subcortical morphology and cerebral white matter energy metabolism in neonates with congenital heart disease. *Sci. Rep.* 8:14057. 10.1038/s41598-018-32288-3 PMC6145929 30232359

[B15] Grady C. L. McIntosh A. R. Craik F. I. (2003). Age-related differences in the functional connectivity of the hippocampus during memory encoding. *Hippocampus* 13 572–586. 10.1002/hipo.10114 12921348

[B16] Jack C. R. Jr. Petersen R. C. Xu Y. O’Brien P. C. Smith G. E. Ivnik R. J. (1998). Rate of medial temporal lobe atrophy in typical aging and Alzheimer’s disease. *Neurology* 51 993–999. 10.1212/wnl.51.4.993 9781519 PMC2768817

[B17] Janmey P. A. Xian W. Flanagan L. A. (1999). Controlling cytoskeleton structure by phosphoinositide-protein interactions: phosphoinositide binding protein domains and effects of lipid packing. *Chem. Phys. Lipids* 101 93–107. 10.1016/s0009-3084(99)00058-4 10810928

[B18] Jones D. R. Varela-Nieto I. (1999). Diabetes and the role of inositol-containing lipids in insulin signaling. *Mol. Med.* 5 505–514. 10.1007/bf03401978 10501653 PMC2230454

[B19] Kakimoto A. Ito S. Okada H. Nishizawa S. Minoshima S. Ouchi Y. (2016). Age-related sex-specific changes in brain metabolism and morphology. *J. Nucl. Med.* 57 221–225. 10.2967/jnumed.115.166439 26609179

[B20] Kizu O. Yamada K. Ito H. Nishimura T. (2004). Posterior cingulate metabolic changes in frontotemporal lobar degeneration detected by magnetic resonance spectroscopy. *Neuroradiology* 46 277–281. 10.1007/s00234-004-1167-5 14991255

[B21] Kocevar G. Stamile C. Hannoun S. Roch J. A. Durand-Dubief F. Vukusic S. (2017). Weekly follow up of acute lesions in three early multiple sclerosis patients using MR spectroscopy and diffusion. *J. Neuroradiol.* 45 108–113. 10.1016/j.neurad.2017.06.010 29032126

[B22] Libon D. J. Bogdanoff B. Cloud B. S. Skalina S. Giovannetti T. Gitlin H. L. (1998). Declarative and procedural learning, quantitative measures of the hippocampus, and subcortical white alterations in Alzheimer’s disease and ischaemic vascular dementia. *J. Clin. Exp. Neuropsychol.* 20 30–41. 10.1076/jcen.20.1.30.1490 9672817

[B23] Lim K. O. Zipursky R. B. Murphy G. M. Jr. Pfefferbaum A. (1990). In vivo quantification of the limbic system using MRI: effects of normal aging. *Psychiatry Res.* 35 15–26. 10.1016/0925-4927(90)90005-q 2367609

[B24] Marsland A. L. Gianaros P. J. Kuan D. C. Sheu L. K. Krajina K. Manuck S. B. (2015). Brain morphology links systemic inflammation to cognitive function in midlife adults. *Brain Behav. Immun.* 48 195–204. 10.1016/j.bbi.2015.03.015 25882911 PMC4508197

[B25] Morey R. A. Petty C. M. Xu Y. Hayes J. P. Wagner H. R. Lewis D. V. (2009). A comparison of automated segmentation and manual tracing for quantifying hippocampal and amygdala volumes. *Neuroimage* 45 855–866. 10.1016/j.neuroimage.2008.12.033 19162198 PMC2714773

[B26] O’Shea D. M. Fieo R. Woods A. Williamson J. Porges E. Cohen R. (2018). Discrepancies between crystallized and fluid ability are associated with frequency of social and physical engagement in community dwelling older adults. *J. Clin. Exp. Neuropsychol.* 40 963–970. 10.1080/13803395.2018.1452195 29569517

[B27] Pacheco J. Goh J. O. Kraut M. A. Ferrucci L. Resnick S. M. (2015). Greater cortical thinning in normal older adults predicts later cognitive impairment. *Neurobiol. Aging* 36 903–908. 10.1016/j.neurobiolaging.2014.08.031 25311277 PMC4315712

[B28] Pagani M. M. Giuliani A. Oberg J. Carli F. Morbelli S. Girtler N. (2017). Progressive disgregation of brain networking from normal aging to Alzheimer’s disease. independent component analysis on FDG-PET data. *J. Nucl. Med.* 58 1132–1139. 10.2967/jnumed.116.184309 28280223

[B29] Paul E. J. Larsen R. J. Nikolaidis A. Ward N. Hillman C. H. Cohen N. J. (2016). Dissociable brain biomarkers of fluid intelligence. *Neuroimage* 137 201–211. 10.1016/j.neuroimage.2016.05.037 27184204

[B30] Penner J. Wells J. L. Borrie M. J. Woolmore-Goodwin S. M. Bartha R. (2015). Reduced N-acetylaspartate to creatine ratio in the posterior cingulate correlates with cognition in Alzheimer’s disease following four months of rivastigmine treatment. *Dement. Geriatr. Cogn. Disord.* 39 68–80. 10.1159/000367685 25358336

[B31] Plows J. F. Budin F. Andersson R. A. Mills V. J. Mace K. Davidge S. T. (2017). The effects of Myo-inositol and B and D vitamin supplementation in the db/+ mouse model of gestational diabetes mellitus. *Nutrients* 9:141. 10.3390/nu9020141 28212289 PMC5331572

[B32] Porges E. C. Woods A. J. Edden R. A. Puts N. A. J. Harris A. D. Chen H. (2017a). Frontal gamma-aminobutyric acid concentrations are associated with cognitive performance in older adults. *Biol. Psychiatry Cogn. Neurosci. Neuroimaging* 2 38–44. 10.1016/j.bpsc.2016.06.004 28217759 PMC5312683

[B33] Porges E. C. Woods A. J. Lamb D. G. Williamson J. B. Cohen R. A. Edden R. A. E. (2017b). Impact of tissue correction strategy on GABA-edited MRS findings. *Neuroimage* 162 249–256. 10.1016/j.neuroimage.2017.08.073 28882635 PMC5705271

[B34] Ramadan S. Lin A. Stanwell P. (2013). Glutamate and glutamine: a review of in vivo MRS in the human brain. *NMR Biomed.* 26 1630–1646. 10.1002/nbm.3045 24123328 PMC3849600

[B35] Rodriguez-Aranda C. Sundet K. (2006). The frontal hypothesis of cognitive aging: factor structure and age effects on four frontal tests among healthy individuals. *J. Genet. Psychol.* 167 269–287. 10.3200/gntp.167.3.269-287 17278416

[B36] Schmitz B. Wang X. Barker P. B. Pilatus U. Bronzlik P. Dadak M. (2018). Effects of aging on the human brain: a proton and phosphorus MR spectroscopy study at 3T. *J. Neuroimaging* 28 416–421. 10.1111/jon.12514 29630746

[B37] Seider T. R. Fieo R. A. O’Shea A. Porges E. C. Woods A. J. Cohen R. A. (2016). Cognitively engaging activity is associated with greater cortical and subcortical volumes. *Front. Aging Neurosci.* 8:94. 10.3389/fnagi.2016.00094 PMC4852201 27199740

[B38] Shewan A. Eastburn D. J. Mostov K. (2011). Phosphoinositides in cell architecture. *Cold Spring Harb. Perspect. Biol.* 3:a004796. 10.1101/cshperspect.a004796 21576256 PMC3140688

[B39] Singhal N. K. Huang H. Li S. Clements R. Gadd J. Daniels A. (2017). The neuronal metabolite NAA regulates histone H3 methylation in oligodendrocytes and myelin lipid composition. *Exp. Brain Res.* 235 279–292. 10.1007/s00221-016-4789-z 27709268 PMC5875422

[B40] Suri S. Emir U. Stagg C. J. Near J. Mekle R. Schubert F. (2017). Effect of age and the APOE gene on metabolite concentrations in the posterior cingulate cortex. *Neuroimage* 152 509–516. 10.1016/j.neuroimage.2017.03.031 28323160 PMC5440729

[B41] Tullberg M. Fletcher E. DeCarli C. Mungas D. Reed B. R. Harvey D. J. (2004). White matter lesions impair frontal lobe function regardless of their location. *Neurology* 63 246–253. 10.1212/01.wnl.0000130530.55104.b5 15277616 PMC1893004

[B42] Valenzuela M. J. Jones M. Wen W. Rae C. Graham S. Shnier R. (2003). Memory training alters hippocampal neurochemistry in healthy elderly. *Neuroreport* 14 1333–1337. 10.1097/00001756-200307180-00010 12876468

[B43] Voevodskaya O. Poulakis K. Sundgren P. van Westen D. Palmqvist S. Wahlund L. O. (2019). Brain myoinositol as a potential marker of amyloid-related pathology: a longitudinal study. *Neurology* 92 e395–e405. 30610093 10.1212/WNL.0000000000006852 PMC6369900

[B44] Wagner M. Jurcoane A. Hildebrand C. Güresir E. Vatter H. Zanella F. E. (2013). Metabolic changes in patients with aneurysmal subarachnoid hemorrhage apart from perfusion deficits: neuronal mitochondrial injury? *AJNR Am. J. Neuroradiol.* 34 1535–1541. 10.3174/ajnr.a3420 23436053 PMC8051459

[B45] Woods A. J. Cohen R. A. Pahor M. (2013). Cognitive frailty: frontiers and challenges. *J. Nutr. Health Aging* 17 741–743. 10.1007/s12603-013-0398-8 24154645 PMC4471842

[B46] Yin D. Liu W. Zeljic K. Wang Z. Lv Q. Fan M. (2016). Dissociable changes of frontal and parietal cortices in inherent functional flexibility across the human life span. *J. Neurosci.* 36 10060–10074. 10.1523/jneurosci.1476-16.2016 27683903 PMC6705576

[B47] Zhang B. Li M. Sun Z. Z. Zhu B. Yuan L. Wang Y. (2009). Evaluation of functional MRI markers in mild cognitive impairment. *J. Clin. Neurosci.* 16 635–641. 10.1016/j.jocn.2008.07.080 19264490

[B48] Zou H. (2006). The adaptive lasso and its oracle properties. *J. Am. Stat. Assoc.* 101 1418–1429. 10.1198/016214506000000735 12611515

